# Resilience to chronic mild stress-induced anhedonia preserves the ability of the ventral hippocampus to respond to an acute challenge

**DOI:** 10.1007/s00406-022-01470-0

**Published:** 2022-08-26

**Authors:** Paola Brivio, Maria Teresa Gallo, Piotr Gruca, Magdalena Lason, Ewa Litwa, Fabio Fumagalli, Mariusz Papp, Francesca Calabrese

**Affiliations:** 1 Department of Pharmacological and Biomolecular Sciences, Università Degli Studi di Milano, Via Balzaretti 9, 20133 Milan, Italy; 2 Maj Institute of Pharmacology, Polish Academy of Sciences, Krakow, Poland

**Keywords:** Chronic stress, Acute stress, Immediate early genes, Early response genes, Resilience, Vulnerability

## Abstract

Stress is a major precipitating factor for psychiatric disorders and its effects may depend on its duration and intensity. Of note, there are differences in individual susceptibility to stress, with some subjects displaying vulnerability and others showing resistance. Furthermore, the ability to react to stressful-life events can alter the response to a subsequent new stressor. Hence, we investigated whether the vulnerability and resilience to the chronic mild stress (CMS) paradigm, in terms of the hedonic phenotype, are paralleled by a different response when facing a novel acute challenge. Specifically, rats submitted to CMS were stratified based on their sucrose intake into vulnerable (anhedonic rats showing reduce intake of sucrose) and resilient (rats not showing the anhedonic-like behavior) subgroups and then further exposed to an acute restraint stress (ARS). Then, neuronal activation was investigated by measuring the gene expression of early immediate (IEG) genes such as *Arc* and *Cfos* and early response (ERG) genes, such as *Gadd45β*, *Sgk1*, *Dusp1*, and *Nr4a1*, in brain regions that play a crucial role in the stress response. We found that resilient rats preserve the ability to increase ERG expression following the ARS selectively in the ventral hippocampus. Conversely, such ability is lost in vulnerable rats. Interestingly, the recovery from the anhedonic phenotype observed in vulnerable rats after 3 weeks of rest from the CMS procedure also parallels the restoration of the ability to adequately respond to the challenge. In conclusion, these findings support the role of the ventral subregion of the hippocampus in the management of both chronic and acute stress response and point to this brain subregion as a critical target for a potential therapeutic strategy aimed at promoting stress resilience.

**Supplementary Information:**

The online version contains supplementary material available at 10.1007/s00406-022-01470-0.

## Introduction

Coping with stress involves the regulation of different systems, namely, neuroendocrine, autonomic, metabolic, immune, and cardiovascular. The ability to efficiently adapt to daily experiences is defined as resilience, i.e., the capacity of an individual to face negative, social, psychosocial, and biological consequences of chronic stress that would otherwise compromise their general well-being [1].

At preclinical level, the chronic mild stress (CMS) animal model is employed to mimic the variability in the stress response characteristic of the human being [2] and to understand the phenomena set in motion in stress-resilient individuals [3]. Indeed, CMS is a well-established procedure that allows stratifying the animals into two populations based on their ability to feel pleasure during pleasurable activities, named hedonic behavior. Specifically, the stress-resilient subgroup is formed by those rats efficiently able to cope with the negative effects of stress, while the vulnerable subset, instead, suffers from the negative consequences of the stress and thus develops the pathological phenotype.

Even if resilience is now considered an active process [4], research aimed at understanding the underlying molecular mechanisms is, nowadays, sparce.

On this topic, we recently found that resilience to CMS can be partly due to the activation of neuroplastic mechanisms within the ventral hippocampus [5] and to the implementations of specific mitochondrial strategies to face with the negative effects of stress [6].

Here, to step forward, we asked if resilience is a stable condition or if, instead, it can be modified by external events.

To this aim, we evaluated whether the response to a previous chronic stress experience, in terms of vulnerability or resilience, may influence the capability of different brain regions to cope appropriately with a subsequent novel stressor. Moreover, we also evaluated if the normalization of the anhedonic phenotype following a period of washout from CMS influences the normal response to a single session of stress.

Notably, since it has been observed that the response to an acute challenge may be altered if the type of stress has been presented previously [7, 8], rats were exposed to one hour of acute restraint stress (ARS), a procedure that is not included as a stressor in the CMS protocol.

While, as mentioned, the response to a chronic negative environment may lead to different consequences on the basis of the capability of the subject to react to a negative stressor, it has been demonstrated that exposure to acute or mild stress provides neuroprotection and mediates several adaptive functions including enhancing cognitive performance and synaptic functions [9, 10]. Furthermore, environmental stimuli during life may sensitize the brain ability to react to novel challenges and a previous stress history may alter the modulation of different pathways with respect to naïve exposure [11].

The ability of the system to respond was evaluated by measuring the expression of genes promptly induced by acute stimuli, the immediate early genes (IEGs) *Arc* and *Cfos* and the expression of the early response genes (ERGs) *Gadd45β*, *Sgk1*, *Dusp1*, and *Nr4a1* [12–16] in the hippocampus (ventral vHip and dorsal dHip), the amygdala (Amy), and the prefrontal cortex (Pfc)*.* The reason why we decided to focus our attention on these genes relies on the evidence that they have been used as markers of neuronal activity for the study of neuronal circuits involved in several brain functions [17].

The limbic structures we focused on mediate the emotional response and support, through dynamic processes, the neuroplastic mechanisms that allow coping with external challenges. Indeed, the plasticity of these brain areas is fundamental to mediate the mechanisms of adaptation following acute stress exposure and chronic stressful-life events may alter the way through which these brain regions may respond to negative experiences [18].

## Materials and methods

### Animals

Adult male Wistar rats (Charles River, Germany) were brought into the laboratory 1 month before the start of the experiment. Except for the first 10 days after arrival when the animals were housed in groups of 10, they were single housed in standard laboratory conditions: food and water was freely available on a 12-h light/dark, constant temperature (22 ± 2 °C), and humidity (50 ± 5%). All procedures used in this study have conformed to the rules and principles of the 2010/63 European Communities Council Directive and have been approved by the Local Bioethical Committee at the Maj Institute of Pharmacology, Polish Academy of Sciences, Krakow, Poland. All efforts were made to minimize animal suffering, and to reduce the number of animals used and the animal studies comply with the ARRIVE guidelines.

### Stress procedure and behavioral test

After adaptation to the housing conditions, rats were trained to consume 1% sucrose solution as previously described [5] and sucrose consumption was monitored at weekly intervals during the whole experiment.

**Fig. 1:**
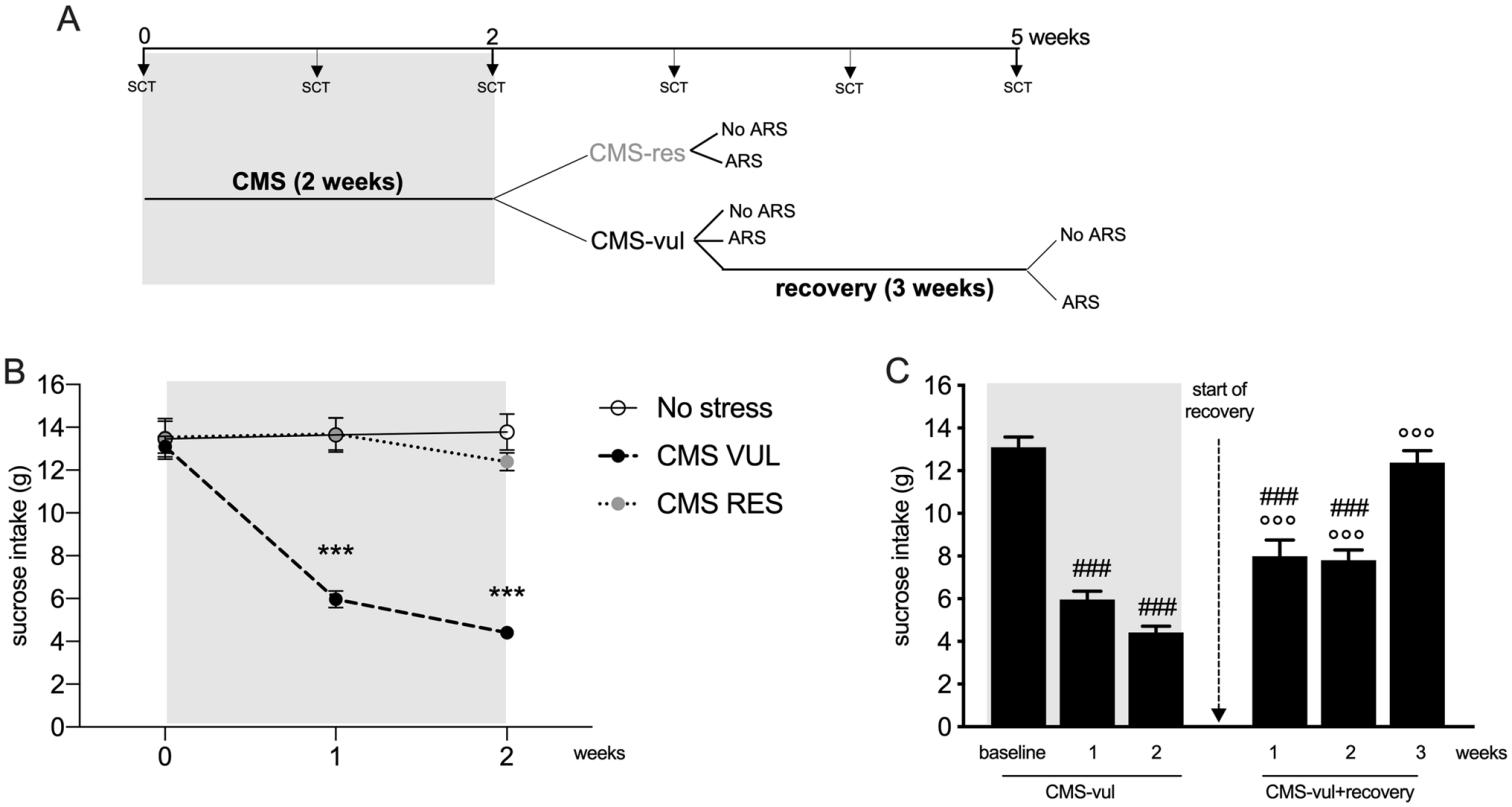
Sucrose consumption test in animals exposed to chronic mild stress (CMS) after a period of recovery from stress. **A** Experimental paradigm; **B** sucrose consumption test (SCT) was performed at weekly intervals during the CMS procedure; **C** SCT performed in vulnerable animals after 3 weeks of recovery from CMS. Data are the mean ± SEM: ***p < 0.001 vs no stress; ^###^*p* < 0.001 vs CMS-vul-baseline, ^°°°^*p* < 0.001 vs CMS VUL-week2 (one-way ANOVA, Fisher’s PLSD)

The experimental paradigm is depicted in Fig. 1A. Briefly, rats were randomly divided into two matched groups: one group was subjected to the CMS procedure for a period of 2 consecutive weeks (see: [19] for details) and the other one was not subjected to the stress protocol. After 2 weeks of CMS, we assigned animals showing the anhedonic phenotype to the CMS-vulnerable (CMS-vul) group and animals that were resilient to the stress protocol to the CMS-resilient (CMS-res group). The cut-off to discriminate among vulnerable and resilient animals was set at 50%. Briefly, CMS-vul consumed around 50% less sucrose solution with respect to their baseline, whereas the CMS-res, despite the CMS procedure, consume similar level of sucrose to the baseline.

At this time point, CMS-vulnerable group was divided into two subgroups. The first one was further divided in two and was immediately exposed or not to the acute restraint stress (ARS), while the second subgroup was left undisturbed in the home cages until the full recovery of the behavioral phenotype (CMS-vul + recovery) and then exposed to one hour of ARS (Fig. 1A).

The animals that were not subjected to the ARS (No ARS) were decapitated 24 h after the final sucrose test whereas the animals belonging to the ARS groups were sacrificed one hour after the ARS procedure. vHip, dHip, Amy and Pfc were dissected from the whole brain according to the plates Paxinos and Watson [20] for the subsequent molecular analyses.

### Blood collection and corticosterone measurement

After the sacrifice, trunk blood was collected in EDTA tubes and centrifuged for 20 min at 3000*g* at 4 °C for the separation of the plasma. Corticosterone plasma levels were assessed with the IBL ELISA kit (Tecan, Italy).

### RNA preparation and gene expression analysis by quantitative real-time PCR

Total RNA was isolated using PureZol RNA isolation reagent (Bio-Rad Laboratories, Italy) according to the manufacturer’s instruction and quantified by spectrophotometric analysis as previously described [21]. Briefly, frozen tissues with PureZol were homogenized with the tissuelyser (Qiagen, Italy) and centrifuged at 12,000 rcf for 20 min at 4 °C. The aqueous phase with RNA was removed, added with isopropyl alcohol, and then centrifuged at 12,000 rcf for 10 min at 4 °C. The pellet of RNA obtained was separated by the supernatant and resuspended in RNase-free water. An aliquot of each sample was then treated with DNase (ThermoFisher scientific, Italy) to avoid DNA contamination. Real-time polymerase chain reaction (RT-PCR) was performed to assess *Arc*, *Cfos*, *Gadd45β*, *Sgk1*, *Dusp1*, and *Nr4a1* mRNA levels. RNA was analyzed by TaqMan qRT-PCR instrument (CFX384 real-time system, Bio-Rad Laboratories, Italy) using the iScriptTM one-step RT-PCR kit for probes (Bio-Rad Laboratories, Italy) (see [22] for details). Samples were run in 384-well formats in triplicate as multiplexed reactions with the normalizing internal control *36B4* (the primers and probes sequences are listed in Table1). A comparative cycle threshold (*C*_t_) method was used to calculate the relative target gene expression.

**Table 1:** (A) Sequences of forward and reverse primers and probes used in real-time PCR analyses and purchased from EurofinsMWG-Operon; (B) probes purchased from Life Technologies, which did not disclose the sequences

(A) Gene	Forward primer	Reverse primer	Probe
*Arc*	GGTGGGTGGCTCTGAAGAAT	ACTCCACCCAGTTCTTCACC	GATCCAGAACCACATGAATGGG
*Cfos*	TCCTTACGGACTCCCCAC	CTCCGTTTCTCTTCCTCTTCAG	TGCTCTACTTTGCCCCTTCTGCC
*Sgk1*	GGTGGGTGGCTCTGAAGAAT	ACTCCACCCAGTTCTTCACC	GATCCAGAACCACATGAATGGG
*Dusp1*	TGTGCCTGACAGTGCAGAAT	ATCTTTCCGGGAAGCATGGT	ATCCTGTCCTTCCTGTACCT
*36b4*	TCAGTGCCTCACTCCATCAT	AGGAAGGCCTTGACCTTTTC	TGGATACAAAAGGGTCCTGG

(B) Gene	Accession number	Assay ID	
*Gadd45β*	BC085337.1	Rn01452530_gI	
*Nr4a1*	BC097313.1	Rn01533237_m1	

### Z-score

*Z* score for each gene has been calculated in the single animals according to the formula

$$Z\mathrm{ score}= \frac{X-\mu }{\sigma }.$$

*X* represents the individual gene expression data of each animal, while $\mu$ and $\sigma$ represent the mean and the standard deviation of the No stress/group.

*Z*-score activation was then calculated by averaging the *Z* score of the IEGs (Fig. 5A–D) and ERGs (Fig. 5E–H) in vHip, dHip, Amy, and Pfc.

### Statistical analysis

The results of the SCT were analyzed with the one-way or two-way analysis of variance (ANOVA) with repeated measures (CMS and time as independent variables and sucrose intake as dependent variable), followed by Fisher’s Protected Least Significant Difference (PLSD), whereas the molecular analyses with the two-way ANOVA (CMS and ARS as independent variables and molecular targets as dependent variables) with PLSD. The results of the two-way ANOVA are listed in the supplementary Tables 1–4. Each experimental group consists of 8 rats. Significance for all tests was assumed for *p* < 0.05. Data are presented as means ± standard error (SEM).

## Results

### Three weeks of rest from CMS leads to a complete recovery of the behavioral phenotype

As we previously observed [5], 2 weeks of CMS induced the development of vulnerable and resilient phenotypes, as indicated by the significant reduction of sucrose intake after 1 (− 56%, *p* < 0.001 vs No stress) and 2 (− 68%, *p* < 0.001 vs No stress) weeks of CMS only in a subgroup of stressed animals that are, therefore, named vulnerable (CMS-vul) (Fig. 1B).

Vulnerable animals took 3 weeks to fully recover from the pathological phenotype (Fig. 1C). Indeed, we observed that starting from the first week after the end of CMS exposure, animals showed a statistically significant increase in the sucrose intake (1 week: + 60%, *p* < 0.001 vs CMS-vul/2 weeks; 2 weeks: + 57%, *p* < 0.001 vs CMS-vul/2 weeks) and, interestingly, 3 weeks of recovery from stress are enough to completely recover from the anhedonic phenotype (+ 148%, *p* < 0.001 vs CMS-vul/2 weeks). Accordingly, at the last time point, sucrose intake of the CMS-vul + recovery group is not statistically different from the baseline, whereas, after 1 and 2 weeks of recovery, sucrose intake is significantly lower in comparison to the vulnerable group at the beginning of the experiment (week1 recovery: − 38%, *p* < 0.001 vs CMS-vul/baseline; week2 recovery: -40%, *p* < 0.001 vs CMS-vul/baseline).

### The IEGs *Arc* and *Cfos* are upregulated by ARS independently from the behavioral phenotype

To map the neuronal responsiveness of brain areas involved in the acute stress response, we measured the expression levels of the immediate early genes *Arc* and *Cfos* in vHip, dHip, Amy, and Pfc.

**Fig. 2:**
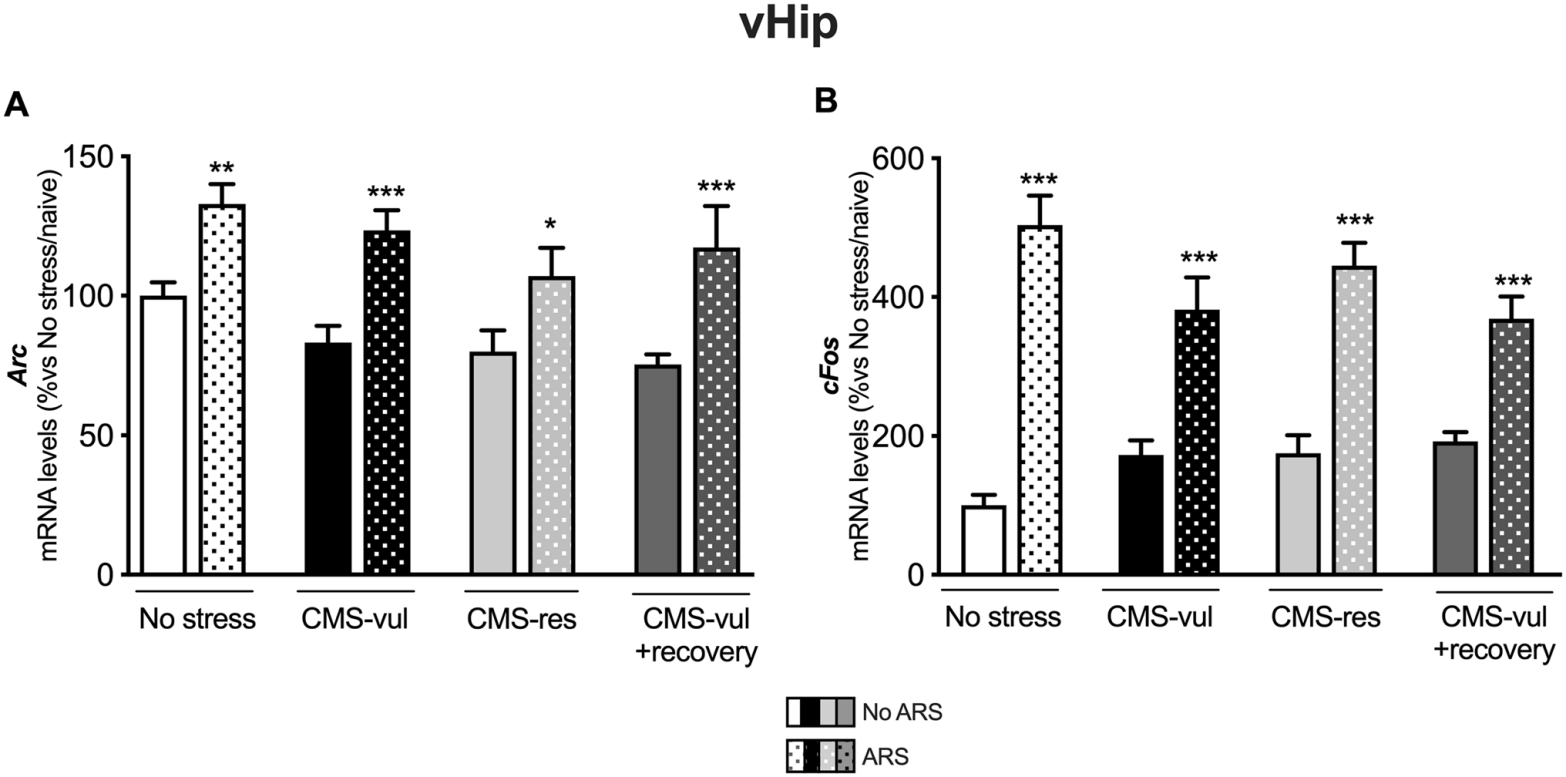
Analysis of *Arc* and *Cfos* mRNA levels in the ventral hippocampus (vHip) of chronically stressed rats exposed to 1 h of acute restraint stress (ARS). The data are the mean ± SEM: *p < 0.05, **p < 0.01, ***p < 0.001 vs no ARS of the same condition (no stress, CMS-vul, CMS-res, and CMS-vul + recovery) (two-way ANOVA, Fisher’s PLSD)

We observed that, in the vHip of non-stressed animals, both *Arc* (Fig. 2A) and *Cfos* (Fig. 2B) are increased by ARS. Of note, the exposure to CMS did not influence this activation, since both the IEGs were upregulated by ARS (Fig. 2) in all animals that underwent the CMS procedure (CMS-vul, CMS-res, and CMS-vul + recovery).

Actually, we observed a similar effect due to ARS in the dHip, Amy, and Pfc (supplementary table 5B–D). Interestingly, we found an increased expression of both *Arc* and *Cfos* in the Pfc of resilient rats and an upregulation of *Arc* in dHip and of *Cfos* in Amy and Pfc of vulnerable rats after the recovery period (supplementary table 5B–D).

### Corticosterone plasma levels are affected by chronic and acute stress exposure

**Fig. 3:**
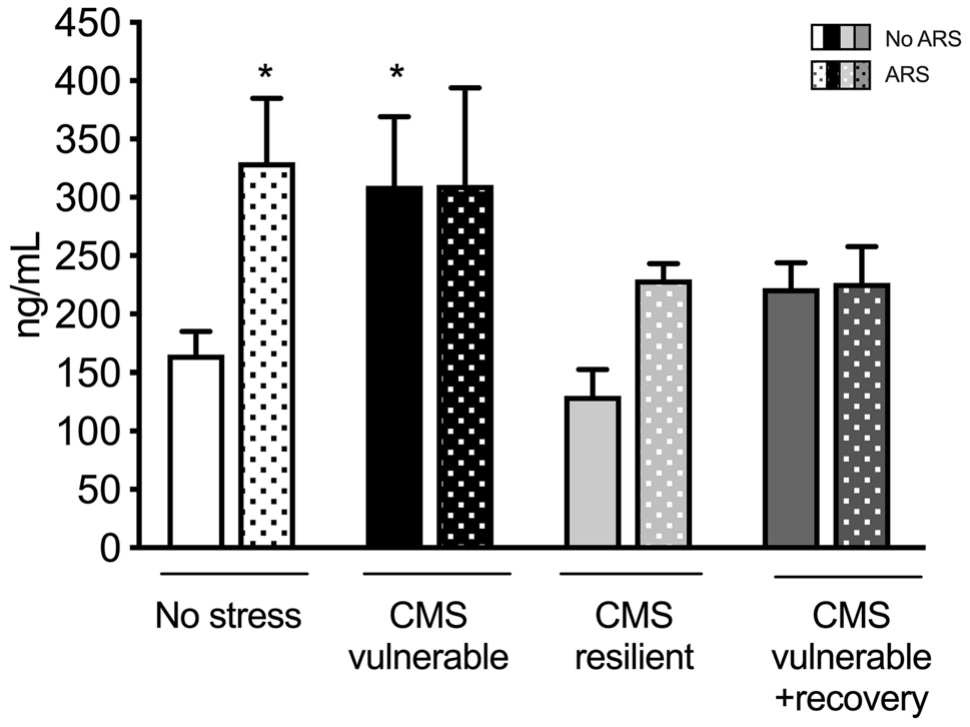
Analysis of corticosterone plasma levels in chronically stressed rats exposed to 1 h of acute restraint stress (ARS). The data are the mean ± SEM: *p < 0.05 vs no ARS of the same condition (no stress, CMS-vul, CMS-res, and CMS-vul + recovery), ^#^*p* < 0.05 vs no stress/no ARS (two-way ANOVA, Fisher’s PLSD)

Given the fundamental role of the HPA-axis activity in stress response [9], we measured corticosterone (CORT) plasma levels and, as previously demonstrated [5], we observed that CMS elevated levels of circulating CORT selectively in vulnerable animals (+ 88%, *p* < 0.05 vs No stress/No ARS), whereas this modulation is observed neither in resilient nor in vulnerable + recovery groups. Moreover, in line with the notion that acute stressor leads to an enhancement of the HPA-axis activity, we found that ARS increased levels of CORT in non-stressed animals (+ 100%, *p* < 0.05 vs No stress/No ARS) as well as in resilient rats (+ 76%, *p* > 0.05 vs No stress/No ARS) even if the augmentation in the latter group did not reach the statistical significance probably due to the complexity of the experimental settings (Fig. 3).

### The ability of the vHip to deal with a novel challenge in terms of ERGs is preserved selectively in resilient animals

We then focused on the expression of ERGs, also known as glucocorticoid responsive genes, namely *Gadd45β, Sgk1*, *Dusp1*, and *Nr4a1*, in a brain region-specific manner, to investigate whether the release of CORT could influence the transcription of these genes strictly dependent from glucocorticoids.

**Fig. 4:**
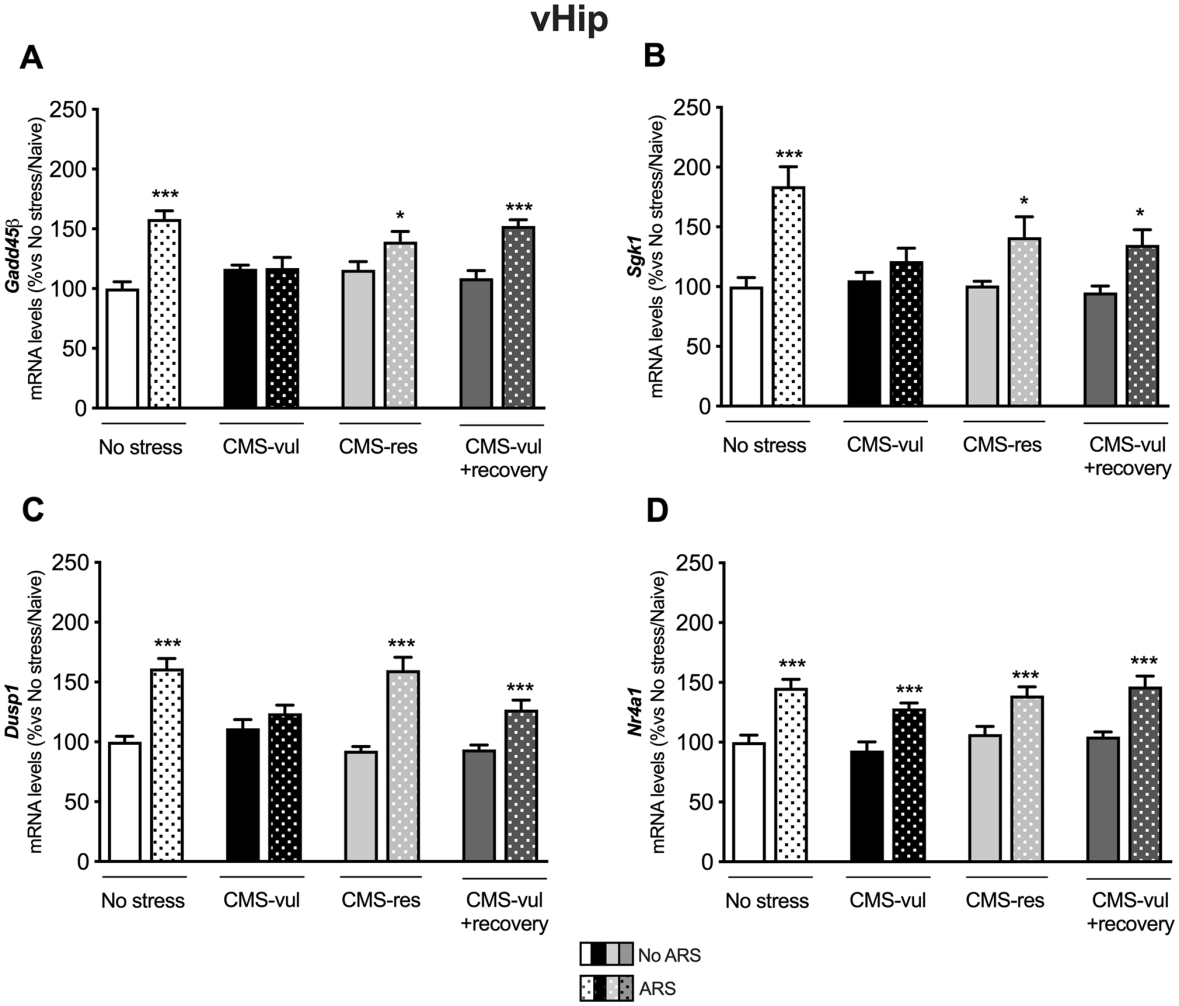
Analysis of *Gadd45β, Sgk1, Dusp1, and Nr4a1* mRNA levels in the ventral hippocampus (vHip) of chronically stressed rats exposed to 1 h of acute restraint stress (ARS). The data are the mean ± SEM: the data are the mean ± SEM: *p < 0.05, ***p < 0.001 vs no ARS of the same condition (no stress, CMS-vul, CMS-res, and CMS-vul + recovery) (two-way ANOVA, Fisher’s PLSD)

In vHip, we found that *Gadd45β* mRNA levels (Fig. 4A) are enhanced by ARS in non-stressed, in CMS-res, in CMS-vul + recovery groups but not in CMS-vul animals. Similarly, ARS induced a similar modulation of *Sgk1* and *Dusp1* (Fig. 4B,C, respectively), with their mRNA levels being upregulated in unstressed, in resilient as well as in recovery group, whereas this effect was completed blunted in vulnerable animals.

Differently, as shown in Fig. 4D, we observed that *Nr4a1* gene expression is increased by ARS regardless of the behavioral phenotype.

By contrast, we observed an overall upregulation of the ERGs expression due to the acute challenge in all the experimental settings in dHip, Amy, and Pfc, as shown in supplementary Table 6B–D.

Of note, resilient rats showed an increased expression of *Gadd45β* gene expression in Pfc, Amy, and dHip and of *Dusp1 and Nr4a1* mRNA levels specifically in Pfc (supplementary table 6B–D). Finally, in the Pfc, *Dusp1* and *Nr4a1* mRNA levels were upregulated in vulnerable animals that recovered from CMS (supplementary table 6 D).

### *Z*-score activation of the ERGs highlights the role of vHip in the susceptibility to stress

**Fig. 5:**
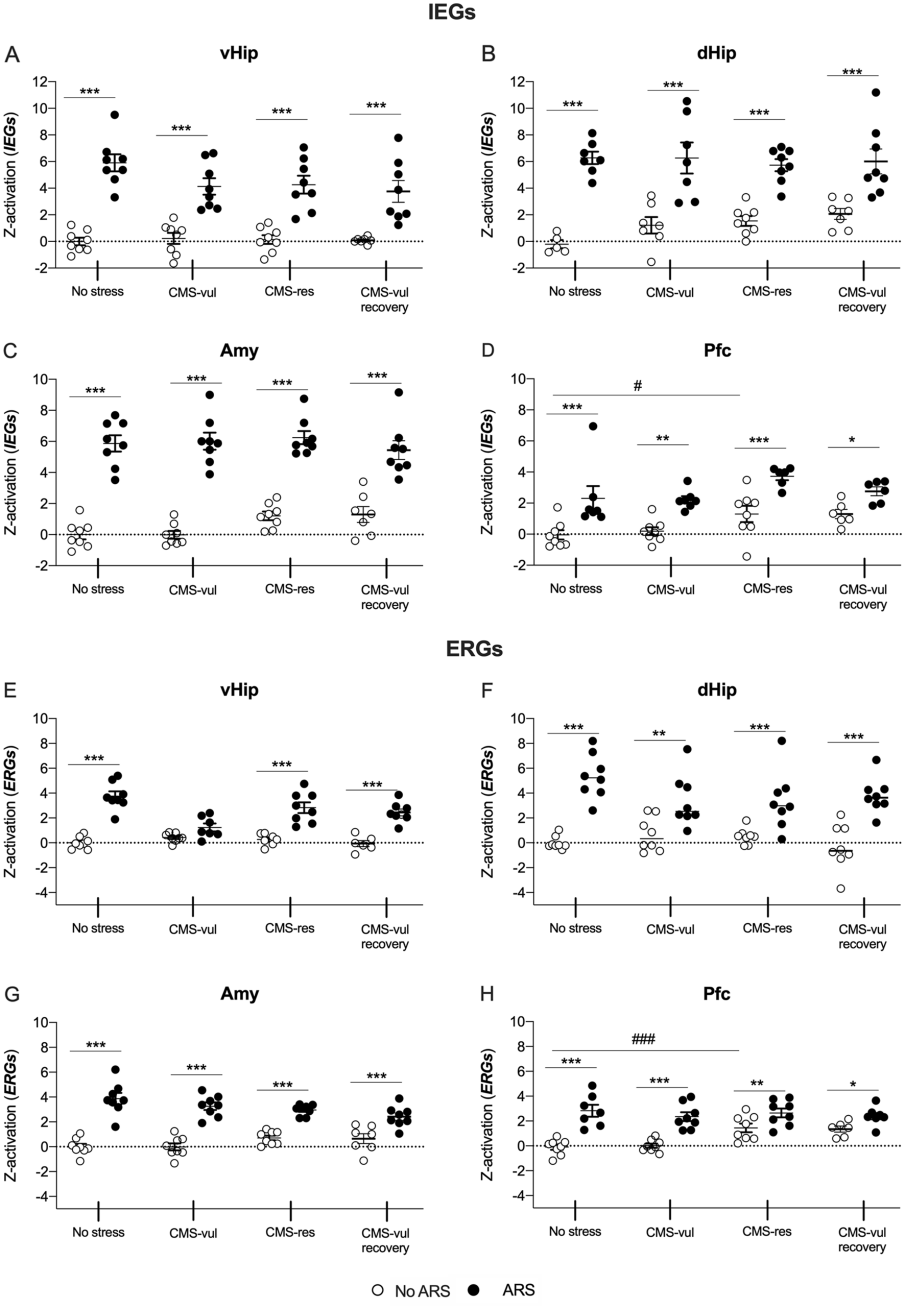
Z-score activation of the IEGs and ERGs in the ventral hippocampus (vHip) (**A**–**E**), dorsal hippocampus (dHip) (**B**–**F**), amygdala (Amy) (**C**–**G**), and prefrontal cortex (Pfc) (**D**–**H**) of chronically stressed rats exposed to 1 h of acute restraint stress (ARS). The data are the mean ± SEM: the data are the mean ± SEM: *p < 0.05, **p < 0.01, ***p < 0.001 vs no ARS of the same condition (no stress, CMS-vul, CMS-res, and CMS-vul + recovery), ^#^*p* < 0.05, ^###^*p* < 0.001 vs no stress/no ARS (two-way ANOVA, Fisher’s PLSD)

We calculated the Z-score activation in each brain region to get a combined overview of the modulation of the IEGs (Fig. 5A–D) and ERGs (Fig. 5E–H) following ARS.

We found a significant *Z-*score activation of the IEGs due to ARS, regardless of the behavioral phenotype, in all the brain regions examined (Fig. 5A–D). Moreover, in Pfc (Fig. 5D), we observed an effect of the stress exposure per sè, with an increased *Z*-score activation in resilient/No ARS animals in comparison to the non-stressed counterpart.

On the contrary, the *Z*-score activation of the ERGs highlighted that 2 week of CMS affected the ability of the vHip to deal with ARS (Fig. 5E) selectively in vulnerable animals. By contrast, in dHip (Fig. 5F), Amy (Fig. 5G), and Pfc (Fig. 5H), Z-score activation indicated that, despite stress exposure, these brain areas were not impaired in their ability to mount a stress response.

Finally, as observed for the IEGs (Fig. 5D), the significant *Z*-score activation of the ERGs in the Pfc (Fig. 5H) of resilient animals suggests that the Pfc activates mechanisms of resilience to face the negative effects of the CMS procedure.

## Discussion

In this study, we demonstrated that resilience is accompanied by changes in the expression levels of IEGs and ERGs mainly in the prefrontal cortex and by the ability of the ventral hippocampus to face a novel challenge, a function that is instead impaired in the subpopulation of animals showing the depressive-like behavior.

According to our previous results [5, 19], here, we confirmed that anhedonia, a core symptom of depression in humans [23], was developed only in a part of animals exposed to the CMS paradigm. Indeed, approximately 30% of stressed animals displayed resilience to the stress protocol.

Moreover, in line with the finding that spontaneous recovery from the depressive-like behavior induced by 6 weeks of CMS was achieved after 4 weeks of rest [24], we showed that vulnerable animals took 3 weeks of washout to completely recover the pathological phenotype.

After the behavioral characterization in terms of hedonic phenotype, we investigated the response of vulnerable and resilient animals to a novel acute stressor by focusing on neuronal activation to explore whether previous stress exposure may have affected their ability to deal with a new challenge.

As of IEGs, we found that, regardless of stress exposure, ARS enhanced the expression of *Arc* and *Cfos* in all the limbic structures investigated, implicating that CMS does not interfere with the activation of these brain structures and suggesting that the responsiveness of these regions was retained, independently from the hedonic phenotype, to promote neuronal mechanisms following external stimuli. In line, we previously found that both ARS and acute forced swim stress exposure [10, 25–27], as well as cognitive demanding task [19], led to an overexpression of the IEGs in unstressed animals in different brain regions; notably, in the Pfc, such modulation was still present after a period of washout following 4 weeks of chronic restraint stress [27].

The hallmark of the stress response is the activation of the HPA axis and the effects of acute and chronic stressors on the axis activity are well described by the inverted U-shaped dose–response curve of glucocorticoids [9]. In particular, activation of the HPA axis and the consequent increase of hormone release are associated with both single and prolonged stress, whereas the outcomes are very different with short and moderate stress being “positive” while chronic stimuli resulting in maladaptive consequences. On these bases, the elevated levels of CORT, already observed in our previous study [5], due to chronic stress have a “negative” impact and are related to the vulnerability to CMS, whereas the transient increase observed following the ARS in no stress and resilient animals may be related to the physiological activation of the HPA axis. Moreover, even if, after the recovery period, the hormone levels almost return to the basal levels, the expected activation induced by ARS exposure is completely absent indicating that CMS induced long-lasting effects.

Afterward, we measured the expression of a series of genes, *Gadd45β*, *Sgk1*, *Dusp1*, and* Nr4a1*, which could be clustered as early response genes. Indeed, the ERGs are rapidly transcribed following acute challenges and respond to HPA-axis activity [12–15].

In the vHip, we found that *Gadd45β*, *Sgk1*, *Dusp1* were enhanced by ARS not only in the control group but also in animals that were resilient to CMS. By contrast, we did not observe this upregulation in CMS-vul group, whereas the vulnerable rats that recovered from the pathological phenotype were still able to mount such response.

On the contrary, in line with the notion that Nr4a1 is highly expressed when the levels of circulating corticosterone are elevated [13], its expression was enhanced by ARS in all the experimental groups, including the vulnerable ones.

Moreover, the physiological recovery from CMS at behavioral level was paralleled by a restored ability to deal with challenging conditions measured as proper response in terms of ERGs following the novel acute stressor. Nevertheless, as mentioned, ARS did not enhance circulating levels of CORT in vulnerable animals following the recovery period, suggesting that the regulation of ERGs expression is complex and involves different and independent systems, which will be further investigated in future studies.

The *Z*-score activation following ARS supports all these results, by highlighting the direction of genes’ regulation in the brain areas considered and strengthening the finding that chronic stress induced functional alterations in vHip in our experimental settings.

The fact that resilient rats exhibited a control-like response to ARS, together with evidence highlighting the importance of neurogenesis [28, 29], neuroplasticity [5], and also the mitochondrial activity [6], further supports the concept that the ventral subregion of the hippocampus is extremely involved in the response to CMS.

Accordingly, we have recently demonstrated that the novel antidepressant drug vortioxetine targeted specifically the vHip by modulating neuronal plasticity following an acute stressor [26].

As observed for the IEGs, we found a different pattern in dHip, Amy, and Pfc, with ARS increased mRNA levels of almost all the genes investigated, regardless of the behavioral susceptibility to stress, pointing out that these limbic structures were not impaired in their ability to be promptly activated following novel stimuli.

Of note, in Pfc, we found an upregulation of the IEGs and *Gadd45β*, *Dusp1*, *Nr4a1* in resilient/No ARS animals in comparison to the unstressed/No ARS counterpart, possibly suggesting that the overexpression of these genes may contribute to promote the mechanisms of resilience. Consistent with this finding, it has been shown that mice resilient to the chronic predator or social defeat stress had a greater degree of *Cfos* expression in the glutamatergic neurons of the Pfc [30, 31], thus indicating that the enhanced neuronal activation in No ARS animals might represent a pro-resilience adaptation. In line, the Pfc has been associated with stressor resistance [32] and it has been shown that this brain region, through the enhancement of neuroplastic mechanisms, is involved in stress-coping strategies to promote resilience [33].

A potential limitation of our findings derives by the evidence that we primarily rely on mRNA data to infer changes in function, i.e., the ability to cope with a further challenge. However, we need to consider that the fast coping to the acute challenge needs to rely on evaluating changes in gene expression as initial responses, whereas changes in the related protein levels would take much longer. Furthermore, as our findings have been collected from male rats, we cannot say whether these results can also be generalized to female rats.

In summary, our results confirm the key role played by the vHip, the hippocampal subregion mainly involved in stress response and in the management of the HPA-axis activity [34], not only in the development of the anhedonic phenotype but also in promoting the mechanisms of resilience both in basal conditions and in response to a novel stress exposure.

## Supplementary Information

Below is the link to the electronic supplementary material.

Supplementary file1 (DOCX 35 kb)

## Data Availability

The datasets generated during and/or analyzed during the current study are available from the corresponding author on reasonable request.
