# Supplementary material for: Resilience to chronic mild stress-induced anhedonia preserves the ability of the ventral hippocampus to respond to an acute challenge
Source: Eur Arch Psychiatry Clin Neurosci. 2022 Aug 26;273(5):1041–50. doi: 10.1007/s00406-022-01470-0 (PMC10359391; doi:10.1007/s00406-022-01470-0)
Supplement: Supplementary file 1 — Supplementary file1 (DOCX 35 kb) [file 406_2022_1470_MOESM1_ESM.docx]

# Supplementary

| SCT | Two-way ANOVA | F (dFn, dFd) | P value |
| --- | --- | --- | --- |
| *2 weeks* | CMS | F (2,62) =50.200 | p=0.000 |
|  | time | F (2,62) =47.700 | p=0.000 |
|  | time X CMS | F (4,62) =51.600 | p=0.000 |
|  | One-way ANOVA | F (dFn, dFd) | P value |
| *vulnerable-recovery* | time X CMS | F (5, 139) =46,062 | p=0.000 |

# Supplementary table 1: One/two-way ANOVA analysis of *the sucrose consumption test with repeated measures.*

| CORT | Two-way ANOVA | F (dFn, dFd) | P value |
| --- | --- | --- | --- |
| *2 weeks* | CMS | F (3,57) =2.917 | p=0.043 |
|  | ARS | F (1,57) =4.780 | p=0.033 |
|  | CMS X ARS | F (3,57) =1.719 | p=0.174 |

# Supplementary table 2: Two-way ANOVA analysis of *the corticosterone plasma levels.*

| IEGs | Two-way ANOVA | F (dFn, dFd) | P value |
| --- | --- | --- | --- |
| vHip | | | |
| *Arc* | CMS | F (1,62) =7.557 | p=0.008 |
|  | ARS | F (1,62) =25.643 | p=0.000 |
|  | CMS X ARS | F (1,62) =0,057 | p=0.813 |
| *Cfos* | CMS | F (1,63) =0.253 | p=0.617 |
|  | ARS | F (1,63) =152.978 | p=0.000 |
|  | CMS X ARS | F (1,63) =13.605 | p=0.000 |
| dHip | | | |
| *Arc* | CMS | F (1,62) =1.279 | p=0.263 |
|  | ARS | F (1,62) =46.879 | p=0.000 |
|  | CMS X ARS | F (1,62) =0.184 | p=0.670 |
| *Cfos* | CMS | F (1,56) =0.982 | p=0.326 |
|  | ARS | F (1,56) =71.349 | p=0.000 |
|  | CMS X ARS | F (1,56) =2.969 | p=0.091 |
| Amy | | | |
| *Arc* | CMS | F (1,62) =1.125 | p=0.293 |
|  | ARS | F (1,62) =110.518 | p=0.000 |
|  | CMS X ARS | F (1,62) =0.463 | p=0.499 |
| *Cfos* | CMS | F (1,63) =0.806 | p=0.373 |
|  | ARS | F (1,63) =203.879 | p=0.000 |
|  | CMS X ARS | F (1,63) =1.361 | p=0.248 |
| Pfc | | | |
| *Arc* | CMS | F (1,54) =4.361 | p=0.042 |
|  | ARS | F (1, 54) =49.042 | p=0.000 |
|  | CMS X ARS | F (1, 54) =0.223 | p=0.639 |
| *Cfos* | CMS | F (1, 50) =13.110 | p=0.001 |
|  | ARS | F (1, 50) =10.238 | p=0.002 |
|  | CMS X ARS | F (1, 50) =0.165 | p=0.687 |
| ERGs | Two-way ANOVA | F (dFn, dFd) | P value |
| vHip | | | |
| *Gadd45b* | CMS | F (1,63) =0.472 | p=0.495 |
|  | ARS | F (1,63) =45.706 | p=0.000 |
|  | CMS X ARS | F (1,63) =8.808 | p=0.004 |
| *Sgk1* | CMS | F (1,63) =8.398 | p=0.005 |
|  | ARS | F (1,63) =43.058 | p=0.000 |
|  | CMS X ARS | F (1,63) =8.697 | p=0.005 |
| *Dusp1* | CMS | F (1,63) =3.741 | p=0.058 |
|  | ARS | F (1,63) =57.788 | p=0.000 |
|  | CMS X ARS | F (1,63) =3.235 | p=0.077 |
| *Nr4a1* | CMS | F (1,59) =0.299 | p=0.587 |
|  | ARS | F (1,59) =56.048 | p=0.000 |
|  | CMS X ARS | F (1,59) =0.496 | p=0.484 |
| dHip | | | |
| *Gadd45b* | CMS | F (1,63) =0.607 | p=0.439 |
|  | ARS | F (1,63) =53.176 | p=0.000 |
|  | CMS X ARS | F (1,63) =2.598 | p=0.112 |
| *Sgk1* | CMS | F (1,63) =7.272 | p=0.009 |
|  | ARS | F (1,63) =39.025 | p=0.000 |
|  | CMS X ARS | F (1,63) =3.648 | p=0.061 |
| *Dusp1* | CMS | F (1,63) =0.017 | p=0.896 |
|  | ARS | F (1,63) =50.278 | p=0.000 |
|  | CMS X ARS | F (1,63) =4.765 | p=0.033 |
| *Nr4a1* | CMS | F (1,62) =1.756 | p=0.190 |
|  | ARS | F (1,62) =34.873 | p=0.000 |
|  | CMS X ARS | F (1,62) =0.180 | p=0.673 |
| Amy | | | |
| *Gadd45b* | CMS | F (1,63) =1.477 | p=0.229 |
|  | ARS | F (1,63) =72.132 | p=0.000 |
|  | CMS X ARS | F (1,63) =5.547 | p=0.022 |
| *Sgk1* | CMS | F (1,63) =6.363 | p=0.014 |
|  | ARS | F (1,63) =39.603 | p=0.000 |
|  | CMS X ARS | F (1,63) =8.424 | p=0.005 |
| *Dusp1* | CMS | F (1,63) =0.834 | p=0.365 |
|  | ARS | F (1,63) =68.774 | p=0.000 |
|  | CMS X ARS | F (1,63) =2.606 | p=0.112 |
| *Nr4a1* | CMS | F (1,62) =0.299 | p=0.587 |
|  | ARS | F (1,62) =113.920 | p=0.000 |
|  | CMS X ARS | F (1,62) =0.627 | p=0.432 |
| PFC | | | |
| *Gadd45b* | CMS | F (1,54) =5.073 | p=0.029 |
|  | ARS | F (1,54) =52.389 | p=0.000 |
|  | CMS X ARS | F (1,54) =0.275 | p=0.603 |
| *Sgk1* | CMS | F (1,54) =1.008 | p=0.320 |
|  | ARS | F (1,54) =11.647 | p=0.001 |
|  | CMS X ARS | F (1,54) =1.527 | p=0.222 |
| *Dusp1* | CMS | F (1,55) =3.043 | p=0.087 |
|  | ARS | F (1,55) =11.752 | p=0.001 |
|  | CMS X ARS | F (1,55) =0.481 | p=0.491 |
| *Nr4a1* | CMS | F (1,58) =3.644 | p=0.061 |
|  | ARS | F (1,58) =42.043 | p=0.000 |
|  | CMS X ARS | F (1,58) = 1.997 | p=0.163 |

# Supplementary table 3: Two-way ANOVA Analysis of *IRGs and ERGs* mRNA levels in the ventral hippocampus (vHip), dorsal hippocampus (dHip), amygdala (Amy) and prefrontal cortex (Pfc) of chronically stressed rats exposed to 1 hour of acute restraint stress (ARS).

| IEGsZ-activation | Two-way ANOVA | F (dFn, dFd) | P value |
| --- | --- | --- | --- |
| vHip | CMS | F (1,62) =1.350 | p=0.2678 |
|  | ARS | F (1,62) =132 | p=0.000 |
|  | CMS X ARS | F (1,62) =1.770 | p=0.1639 |
| dHip | CMS | F (1,56) =0.693 | p=0.5609 |
|  | ARS | F (1,56) =104 | p=0.000 |
|  | CMS X ARS | F (1,56) =1.300 | p=0.2846 |
| Amy | CMS | F (1,62) =1.360 | p=0.2643 |
|  | ARS | F (1,62) =278 | p=0.000 |
|  | CMS X ARS | F (1,62) =1.840 | p=0.61508 |
| Pfc | CMS | F (1,55) =5.07 | p=0.0040 |
|  | ARS | F (1,55) =47.3 | p=0.000 |
|  | CMS X ARS | F (1,55) =0.491 | p=0.6899 |
| ERGsZ-activation | Two-way ANOVA | F (dFn, dFd) | P value |
| vHip | | | |
| vHip | CMS | F (1,59) =4.900 | p=0.004 |
|  | ARS | F (1, 59) =135 | p=0.000 |
|  | CMS X ARS | F (1, 59) =8.840 | p=0.000 |
| dHip | CMS | F (1,63) =1 | p=0.084 |
|  | ARS | F (1,63) =85.700 | p=0.000 |
|  | CMS X ARS | F (1,63) =2.330 | p=0.398 |
| Amy | CMS | F (1,62) =0.740 | p=0.532 |
|  | ARS | F (1,62) =170 | p=0.000 |
|  | CMS X ARS | F (1,62) =4.940 | p=0.004 |
| Pfc | CMS | F (1,59) =3.2 | p=0.028 |
|  | ARS | F (1,59) =67 | p=0.000 |
|  | CMS X ARS | F (1,59) =3.8 | p=0.014 |

# Supplementary table 4: Two-way ANOVA Analysis of the Z activation of the *IRGs and ERGs* mRNA levels in the ventral hippocampus (vHip), dorsal hippocampus (dHip), amygdala (Amy) and prefrontal cortex (Pfc) of chronically stressed rats exposed to 1 hour of acute restraint stress (ARS).

| IEGs | | No stress | | CMS VUL | | CMS RES | | CMS VUL-REC | |
| --- | --- | --- | --- | --- | --- | --- | --- | --- | --- |
|  |  | No ARS | ARS | No ARS | ARS | No ARS | ARS | No ARS | ARS |
| AvHip | *Arc* | 100±5 | 133±7** | 83±6 | 123±7*** | 80±8 | 107±10* | 75±4 | 117±15*** |
|  | *Cfos* | 100±15 | 504±43*** | 173±21 | 382±47*** | 175±26 | 445±33*** | 192±13 | 369±32*** |
| BdHip | *Arc* | 100±8 | 152±3 *** | 94±6 | 151±8 *** | 114±10 | 159±11 *** | 127±10#§ | 162±10** |
|  | *Cfos* | 100±16 | 470±34 *** | 202±40 | 475±80*** | 189±15 | 422±25*** | 207±20 | 437±57*** |
| CAmy | *Arc* | 100±9 | 226±11*** | 102±9 | 236±18*** | 131±11 | 234±17*** | 126±16 | 220±17*** |
|  | *Cfos* | 100±19 | 451±43*** | 93±12 | 444±29*** | 161±14 | 473±21*** | 186±23#§ | 419±38*** |
| DPfc | *Arc* | 100±18 | 245±35*** | 103±14 | 251±15*** | 209±25### §§ | 321±7** | 143±16 | 259±26** |
|  | *Cfos* | 100±26 | 161±24 | 111±13 | 201±17** | 186±25## § | 298±34** | 222±22### §§ | 265±23 |

# Supplementary table 5: *Arc* and *Cfos* mRNA levels in the ventral (vHip) (A) and dorsal hippocampus (dHip) (B), the amygdala (Amy) (C) and the prefrontal cortex (Pfc) (D) of chronically stressed rats exposed to 1 hour of acute restraint stress (ARS).

# The data are the mean ± SEM: ^*^ p<0.05, ^**^ p<0.01, ^***^ p<0.001 vs No ARS of the same condition (No stress, CMS-vul, CMS-res, CMS-vul+recovery); ^#^ p<0.05, ^##^p<0.01, ^###^ p<0.001 vs No stress/No ARS, ^§^p<0.05 ^§§^p<0.01 ^§§§^p<0.001 vs CMS-vul/No ARS (two-way ANOVA, Fisher’s PLSD).

| ERGs | | No stress | | CMS VUL | | | CMS RES | | CMS VUL-REC | |
| --- | --- | --- | --- | --- | --- | --- | --- | --- | --- | --- |
|  |  | No ARS | ARS | | No ARS | ARS | No ARS | ARS | No ARS | ARS |
| AvHip | *Gadd45β* | 100±6 | 158±7*** | | 116±3 | 117±9 | 116±7 | 139±8* | 109±7 | 153±5*** |
|  | *Sgk1* | 100±7 | 184±16*** | | 105±7 | 121±11 | 101±4 | 141±17* | 95±5 | 135±13* |
|  | *Dusp1* | 100±5 | 161±8*** | | 111±7 | 120±6 | 92±4 | 160±11*** | 94±4 | 127±8*** |
|  | *Nr4a1* | 100±6 | 145±7*** | | 93±7 | 128±5*** | 107±6 | 139±7*** | 105±4 | 147±9*** |
| BdHip | *Gadd45β* | 100±4 | 144±3*** | | 109±7 | 134±5** | 123±6## | 141±5* | 104±6 | 144±8*** |
|  | *Sgk1* | 100±2 | 162±12*** | | 97±5 | 129±11* | 93±3 | 119±16 | 93±6 | 133±11** |
|  | *Dusp1* | 100±5 | 158±10*** | | 127±8# | 145±9 | 118±3 | 151±7** | 94±8§§ | 136±5*** |
|  | *Nr4a1* | 100±5 | 135±6** | | 111±6 | 133±7* | 103±7 | 144±8*** | 116±7 | 144±8** |
| CAmy | *Gadd45β* | 100±7 | 170±8*** | | 113±10 | 166±5*** | 128±7# | 165±9** | 128±7# | 158±10* |
|  | *Sgk1* | 100±6 | 152±12*** | | 103±6 | 124±8* | 106±4 | 128±5* | 97±6 | 112±5 |
|  | *Dusp1* | 100±5 | 168±9*** | | 92±6 | 166±10*** | 108±7 | 150±9*** | 114±9 | 136±7 |
|  | *Nr4a1* | 100±8 | 194±13*** | | 91±8 | 186±10*** | 111±5 | 179±10*** | 104±8 | 183±16*** |
| DPfc | *Gadd45β* | 100±10 | 200±22*** | | 96±14 | 235±23*** | 144±10# § | 257±26*** | 139±12 | 226±15** |
|  | *Sgk1* | 100±4 | 126±11** | | 92±3 | 123±6** | 111±7 | 104±7 | 103±4 | 112±9 |
|  | *Dusp1* | 100±7 | 149±14* | | 104±7 | 154±15*** | 143±13# | 150±13 | 148±5# | 153±11 |
|  | *Nr4a1* | 100±13 | 271±20*** | | 141±7 | 263±28** | 185±27# | 263±19* | 197±13# | 277±35** |

# Supplementary table 6: *Gadd45β, Sgk1, Dusp1 and Nr4a1* mRNA levels in the dorsal hippocampus (dHip) (A), the amygdala (Amy) (B) and the prefrontal cortex (Pfc) (C) of chronically stressed rats exposed to 1 hour of acute restraint stress (ARS).

# The data are the mean ± SEM: ^*^ p<0.05, ^**^ p<0.01, ^***^ p<0.001 vs No ARS of the same condition (No stress, CMS-vul, CMS-res, CMS-vul+recovery); ^#^ p<0.05, ^##^ p<0.01 vs No stress/No ARS, ^§^p<0.05 ^§§^p<0.01 vs CMS-vul/No ARS (two-way ANOVA, Fisher’s PLSD).
